# Supplementary material for: Discovery and pharmacophoric characterization of chemokine network inhibitors using phage-display, saturation mutagenesis and computational modelling
Source: Nat Commun. 2023 Sep 16;14:5763. doi: 10.1038/s41467-023-41488-z (PMC10505172; doi:10.1038/s41467-023-41488-z)
Supplement: Supplementary file 4 — Source Data [file 41467_2023_41488_MOESM4_ESM.zip › SourceData/scripts/xNNKs_20230808183658.tar!/xNNKs/analysis/heatmaps/SV_HD2X_valine_heatmap.pdf]

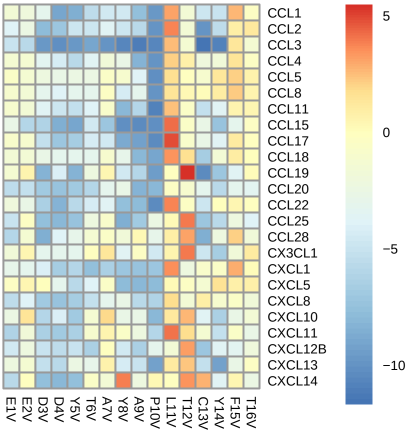

CCL1
CCL2
CCL3
CCL4
CCL5
CCL8
CCL11
CCL15
CCL17
CCL18
CCL19
CCL20
CCL22
CCL25
CCL28
CX3CL1
CXCL1
CXCL5
CXCL8
CXCL10
CXCL11
CXCL12B
CXCL13
CXCL14
E1V
E2V
D3V
D4V
Y5V
T6V
A7V
Y8V
A9V
P10V
L11V
T12V
C13V
Y14V
F15V
T16V
5
0
−5
−10
